# Biomimetic cellulose/calcium-deficient-hydroxyapatite composite scaffolds fabricated using an electric field for bone tissue engineering†

**DOI:** 10.1039/c8ra03657h

**Published:** 2018-06-06

**Authors:** MyoJin Kim, MiJi Yeo, Minseong Kim, GeunHyung Kim

**Affiliations:** Department of Biomechatronic Eng., College of Biotechnology and Bioengineering, Sungkyunkwan University (SKKU) Suwon South Korea gkimbme@skku.edu +82-31-290-7828

## Abstract

Cellulose has been widely used as micro/nanofibers in various applications of tissue regeneration, but has certain limitations for bone regeneration, *e.g.*, low biocompatibility in inducing osteogenesis. In addition, the low processability from the decomposition property before melting can be a significant obstacle to fabricating a required complex structure through a 3D-printing process. Herein, to overcome the low osteogenic activity of pure cellulose, we suggest a new cellulose-based composite scaffold consisting of cellulose and a high weight fraction (70 wt%) of calcium-deficient-hydroxyapatite (CDHA), which was obtained from the hydrolysis of α-tricalcium phosphate. Using biocompatible components, we fabricated a 3D pore-structure controllable composite scaffold consisting of microfibrous bundles through an electrohydrodynamic printing (EHDP) process supplemented with an ethanol bath. To obtain a mechanically stable and repeatable 3D mesh structure, various process parameters (nozzle-to-target distance, electric field strength, flow rate, and nozzle moving speed) were considered. As a control, a mesh structure fabricated using a normal EHDP process and with a similar pore geometry was used. A variety of cellular responses using preosteoblasts (MC3T3-E1) indicate that a CDHA/cellulose composite scaffold provides an efficient platform for inducing significantly high bone mineralization.

## Abstract 2

The fabricated ceramic scaffold showed a layer-by-layered mesh structure entangled with cellulose micro/nanofibers and the bioceramic phase. By varying processing parameters, the unique 3D fibrous mesh-structure could be achieved.

## Graphical abstract

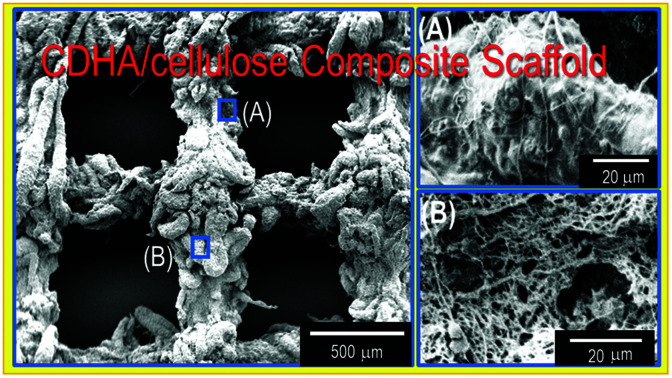

## Introduction

A considerable number of patients with bone defects created through trauma, infection, tumor resection, or skeletal abnormalities can be found worldwide.^1^ As the necessity for bone treatment has increased, research on bone grafting and regeneration is becoming more important. For bone treatment, the grafting of human-derived bone tissue (allograft) has certain limitations in terms of donor supply shortage and a risk of infection.^2^ Therefore, to compensate for these problems, the regeneration of bone tissue using an artificial biomimetic scaffold is preferred, and has become a promising technique in tissue engineering.

A scaffold is an artificial structure that provides a mechanical and physiological support to cells for *in vitro* tissue regeneration and/or *in vivo* implantation. To fabricate a scaffold, its micro- and macro-structures should be designed properly because the structures of the scaffolds affect the adhesion and differentiation of cells.^3,4^ A three-dimensional (3D) scaffold acts as an extracellular matrix (ECM) in which cells originally reside, and the most favorable scaffold has been an ECM-like structure, which is composed of nanofibers.^3,5–7^ In addition, the scaffold should be highly porous to allow for cell ingrowth and efficient mass transport of nutrients, oxygen, growth factors, and waste products.^8^

To design a scaffold, physiological conditions should be met to regenerate various types of tissues. The scaffold must be biocompatible, and the decomposed components must be harmless *in vivo.*^9^ Moreover, properties inducing osteogenic differentiation, such as osteoinductivity and osteoconductivity, should be provided to osteoblasts for realistic bone regeneration. To meet these requirements, a variety of biocompatible materials have been used, including natural polymers such as alginate, collagen, and cellulose; synthetic polymers such as polycaprolactone and poly(lactic-*co*-glycolic acid); and bioceramics such as hydroxyapatite and calcium phosphate;^10–13^ ceramic reinforced biocomposites such as carbon nanotube, graphene and boron nitride nanotube added polymers.^14–16^ Of the natural biopolymers, cellulose is the most widespread polymeric material found in nature, such as in plants and bacteria.^13^ It has high biocompatibility, protein binding sites on its surface, a reasonable mechanical strength, and resistance to breakdown *in vivo.*^17^ The major composition of cellulose fiber has been proven to be biocompatible for both granulation tissue and bone formation. In addition, its fiber has a high density of reactive hydroxyl groups on its surface, which facilitate the immobilization of cell adhesive proteins such as fibronectin.^18^ Therefore, cellulose has emerged as a potential biocompatible material for the fabrication of various scaffolds. However, despite its potential use as a biomedical scaffold, cellulose has not been widely applied in the field of hard tissue regeneration owing to a loss of osteoconductivity.^19,20^ To overcome the shortcoming of osteogenesis for pure cellulose, various composites using bioceramics (hydroxyapatite and calcium phosphate) have been accommodated.^21,22^

In terms of structural formation, pure cellulose cannot be used to directly fabricate a 3D structure consisting of microstruts using a 3D printer because it is easily degraded or decomposed before melting takes place.^23^ Most methods used to fabricate cellulose-based structures apply a low concentration of cellulose as a dispersed phase in blend/composite systems and solvents (acetone).^24–26^ For this reason, the cellulose in solvents has been widely fabricated into electrospun fibers. However, an electrospun mat cannot provide controllable micro/macro-pores owing to densely stacked nanofibers that can block cell migration or ingrowth into the thickness direction of the electrospun mat.^10,27^

To compensate for these limitations, we accommodated an electrohydrodynamic printing (EHDP) process and suggested a new biocomposite structure consisting of cellulose and α-tricalcium phosphate (α-TCP). We recently developed the EHDP process, which uses a charged single jet and target medium (ethanol) for fabricating a 3D pore-size controllable structure consisting of fibrous bundles.^28^ The fabricated structure demonstrates ideal scaffold shapes and shows reasonably high cellular responses owing to the controllable macropores as compared to those of a general 3D-printed structure, when cultured using MC3T3-E1.^28^

Nevertheless, a 3D cellulose structure requires more enhanced osteogenic activities to efficiently regenerate bone tissue. However, as there are much more advantages, we still selected this material as a tissue regenerative agent supplemented with calcium-deficient-hydroxyapatite (CDHA). CDHA is an osteoconductive mineral frequently used as a bone substitute, and in particular, CDHA improves bone formation and suppresses bone resorption.^7,29^ Therefore, we present a cellulose/CDHA composite scaffold fabricated using the EHDP process to efficiently regenerate bone tissue. The cellulose/CDHA scaffold was obtained from the hydrolysis of α-TCP component. The EHDP process is significantly dependent on the processing parameters, applied electric field, the distance between the nozzle and target medium, and the moving speed of the nozzle for the formation of a fibrous bundle structure, and thus we selected the most appropriate processing conditions to obtain a cellulose/ceramic-based bone-mimetic fibrous composite with a high ceramic wt% (70 wt%).^28^ We then investigated various cellular responses affected by a fibrous composite with a macroporous structure; a scaffold fabricated using EHDP (EHDP-scaffold) was compared with a control scaffold (P-scaffold) fabricated through a general EHDP printing process without the use of an ethanol medium as a target. The fabricated composite scaffolds were evaluated based on various cellular responses, the initial cell attachment, fluorescence images, and cell proliferation and differentiation using preosteoblasts (MC3T3-E1).

## Materials & methods

### Materials

In this study, α-TCP was provided by Dr H.-S. Yun (Powder and Ceramics Division, Korea Institute of Materials Science, South Korea), and cellulose acetate (density of 1.3 g cm^−3^, *M*_n_ of 30 000 g mol^−1^) was purchased from Sigma-Aldrich Co. (St. Louis, MO, USA). We used 7 : 3 weight fraction of α-TCP and cellulose mixture by dispersing α-TCP in cellulose solution. This cellulose solution was diluted to 20 wt% with a solvent mixture of acetone (surface tension = 24 mN m^−1^) and dimethylformamide (DMF; surface tension of 37.1 mN m^−1^) (Junsei Chemical Co., Tokyo, Japan) at a 1 : 1 ratio.

### Fabrication of a cellulose/α-TCP composite fibrous scaffold

**Fig. 1:**
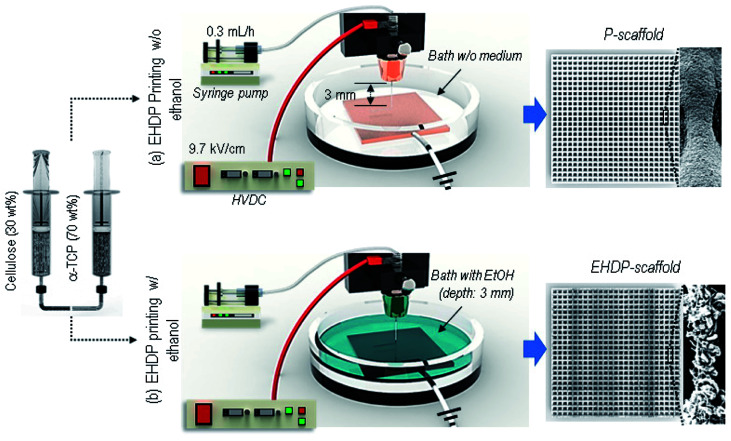
Schematic of the fabrication of 3D scaffolds using an electrohydrodynamic printing (EHDP) process (a) without an ethanol medium to fabricate a scaffold (P-scaffold), and (b) with ethanol to fabricate an EHDP-scaffold.

A scaffold with latticed struts was printed using a three-axis robot moving system (DTR3-2210-T-SG, DASA Robot, South Korea), which controls the moving speed of the nozzle (2.5–15 mm s^−1^) and the distance between the nozzle tip and collector. The collector is a copper plate where the injected solution reaches, and was built into the struts. To make micro/nano-fibrous struts, ethanol (C_2_H_5_OH, *M*_n_ of 46.07 g mol^−1^, surface tension of 22.39 mN m^−1^) was filled as a target solution on a copper plate. We supplied an electric field on a nozzle using a power supply (SHV300RD-50K; Convertech, Seoul, South Korea) within a range of 9.2–11.7 kV cm^−1^, and the ground was connected to the copper plate. As an electrohydrodynamically printed scaffold (EHDP-scaffold), a mixed solution of α-TCP/cellulose is extruded from a syringe pump (KDS 230; KD Scientific, Holliston, MA, USA) through a 21G nozzle (inner diameter of 500 μm) at a 0.3 mL h^−1^ flow rate (Fig. 1). To fabricate a control scaffold, the printed scaffold (P-scaffold) with non-fibrous struts was drawn into the collector not filled with ethanol under the same condition of fabricating EHDP-scaffold (Fig. 1).

All scaffold samples were fabricated under the condition of 17 ± 2 °C temperature and 19 ± 3% humidity. After printing, the samples were washed with distilled water, immersed in PBS for 24 h to induce cementation, and lastly washed again with ethanol, PBS, and distilled water in sequence.

### Characterization of cellulose/α-TCP composite scaffold

To observe the structure and surface topography of the fabricated scaffolds, a scanning electron microscope (SEM, SNE-3000M, SEC, Inc., South Korea) was used. The diameter of the struts and the pore structure were quantitatively measured (*n* = 50) using ImageJ software (National Institute of Health, Bethesda, MD). To quantitatively indicate the pore size of electrospun fibers, the polynomial shapes of individual pores were simplified into ellipses. The diameters were then calculated as (4 × area/π)^1/2^.

To calibrate the weight fraction of calcium-deficient hydroxylapatite (CDHA) after cementation, the α-TCP/cellulose composite scaffold was weighed. The sample was then immersed in acetone for 24 h, which dissolves cellulose. The dissolved sample was air-dried, and its weight was measured.

To measure the protein absorption, we used a bicinchoninic acid (BCA) protein assay (Pierce Kit; Thermo Scientific, Waltham, MA, USA). The P- and EHDP-scaffolds (5 × 5 × 0.5 mm^3^ in size) were immersed in a α-minimum essential medium (α-MEM) and 10% fetal bovine serum (FBS) (Gemini Bio-Products, Calabasas, CA, USA), and incubated at 37 °C for 4, 6, and 24 h. The samples were lysed with 0.1% Triton X-100 and incubated again for 2 h. An aliquot of the lysate (25 μL) was then added to 200 μL of the BCA working reagent. After incubating for 30 min at 37 °C, the optical density was measured at 562 nm using a plate reader. Based on the optical density result, the protein absorption values are obtained using a standard curve as the means ± SD (*n* = 4).

To obtain the porosity of each scaffold, we calculated the mathematical ratio, which is the volume of voids divided by the total volume. The total volume was obtained by multiplying each length of the edge. Here, we obtained the volume of the voids indirectly by subtracting the bulk volume from the total volume, and the bulk volume was calculated based on the weight and density of the composite scaffolds.

To confirm whether α-TCP turns into CDHA after the cementation process, the samples were compared through an X-ray diffraction (XRD; Siemens D500 WAXD, Munich, Germany) analysis using a Scintag automated diffractometer. X-rays were irradiated at 20 mA and 40 kV, with a scan rate of 20–40° 2*θ* and a 0.1° step size throughout this range.

To compare mechanical property of each scaffold, we obtained compressive stress–strain curves and compressive moduli from each scaffold. Compressive stress–strain curve was measured using a universal testing instrument (Top-tech 2000; Chemilab, South Korea) from each scaffold (*n* = 5), with the sample size of 5 × 5 × 2 mm^3^ at compression rate of 0.15 mm s^−1^.

### *In vitro* cell culture

Mouse preosteoblast cells (MC3T3-E1; ATCC, Manassas, VA, USA) at a density of 1 × 10^5^ per specimen were seeded on scaffolds measuring 5 × 5 × 1 mm^3^ in size. The samples were maintained in α-MEM (Life Sciences, Carlsbad, CA, USA) supplemented with 1% antibiotic (antimycotic; Cellgro, Manassas, VA) and 10% fetal bovine serum (FBS; Gemini Bio-Products, Sacramento, CA, USA), and incubated with 5% CO_2_ at 37 °C. The medium was exchanged every second day.

### *In vitro* cellular activities

The cell seeding efficiency and proliferation rate of viable cells were assessed using an MTT assay (Cell Proliferation Kit I; Boehringer Mannheim, Mannheim, Germany). MTT induces mitochondrial dehydrogenases, and cleaves the yellow tetrazolium salt of viable cells, which results in purple formazan crystals. The samples were incubated in the MTT solution for 4 h, and a solubilization solution was added overnight at 37 °C. Using an ELISA reader (EL800; BioTek Instruments, Winooski, VT, USA) at 570 nm, the absorbance was measured after 1, 3, and 7 days of culturing.

For cell viability at days 1, 3, and 7, 0.15 mM calcein AM and 2 mM ethidium homodimer-1 were used to stain the cells for 1 h. A fluorescence microscope (model BXFM-21; Olympus, Tokyo, Japan) was used to capture the images, which revealed a green color for live cells and a red color for dead cells. The numbers of live and dead cells were measured using ImageJ software, and were represented in the cell viability.

Following 7 and 14 days of culturing, the cell nuclei and cytoskeleton were stained using diamidino-2-phenylindole (DAPI) and phalloidin (Invitrogen, Carlsbad, CA), respectively. The images were obtained using a laser-scanning microscope (LSM510; Carl Zeiss, Oberkochen, Germany).

Images of F-actin after 7 days of culturing were used to measure the stretching ratio (SR) of the cell morphology, which was obtained based on the ratio of the longest to shortest axes of the elongated cells. More than 50 cells were measured for circularity using ImageJ software.

A BCA protein assay (Pierce kit; Thermo Scientific) was conducted at days 7 and 14 to measure the total protein content. The scaffolds were rinsed with PBS, and lysed using 1 mL of 0.1% Triton X-100. After 200 μL of a BCA working reagent was added with an aliquot of lysate (25 μL), the mixture was incubated at 37 °C for half an hour. At an absorbance of 570 nm, an enzyme-linked immunosorbent assay reader was used to measure the total protein concentration with a standard curve.

The marker of osteoblast activity, alkaline phosphatase (ALP), was assayed through the release of *p*-nitrophenol from *p*-nitrophenyl phosphate (*p*-NPP) at days 7 and 14. After rinsing the scaffolds seeded with MC3T3-E1 using a phosphate buffer saline (PBS), the samples were incubated for 10 min in a 10 mM Tris buffer (pH 7.5) containing 0.1% Triton X-100. Then, using an ALP kit (procedure no. ALP-10; Sigma), 100 μL of lysate was added to 100 μL of *p*-NPP contained in a 96-well tissue culture plate. Therefore, *p*-NPP was transformed into inorganic phosphate and *p*-nitrophenol. The ALP activity was analyzed using a microplate reader (Spectra III; SLT-Lab Instruments, Salzburg, Austria) at an absorbance of 405 nm.

An Alizarin Red-S (ARS) assay was applied using MC3T3-E1 cells on the scaffolds to show their calcium mineralization. The cells were then fixed in 70% cold ethanol (4 °C) for 1 h. After air drying, the fixed specimens were stained using 40 mM Alizarin Red-S for 1 h, and rinsed three times using tri-distilled water. The samples were immersed and destained in 10% cetylpyridinium chloride in a 10 mM sodium phosphate buffer for 15 min. Optical images were obtained using a microscope, and the ARS optical density was measured at an absorbance of 562 nm.

To analyze the cell adherence morphology and calcium and phosphorus distribution, the samples were fixed using 2.5 wt% glutaraldehyde for 1 h, and rinsed with 50%, 60%, 70%, 80%, 90%, and 100% ethanol for 10 min each. After blocking with hexamethyldisilazane for 4 h, the samples were air-dried to capture SEM images and energy-dispersive spectroscopy (EDS).

### Statistical analysis

All data were obtained from at least three replicates for each sample, and were represented as the means ± standard deviation. Using SPSS software (SPSS, Inc., Chicago, IL), a statistical analysis was applied, and single-factor analyses of variance (ANOVA) were included. The statistical significance was indicated by an asterisk (*) for *P* values < 0.05, and “NS” shows that no significance was found for *P* values > 0.05.

## Results & discussion

### Fabrication of composite scaffolds using EHDP printing process

To attain a desired fibrous strut structure, we used two EHDP printing methods, as described in Fig. 1(a and b). A schematic shows the fabrication process, from preparing the solution to obtaining the final composite scaffolds (P-scaffold and EHDP-scaffold); the printing systems consist of four parts: a syringe pump, power supplier, three-axis robot moving system, and grounded target. A syringe was fixed to the syringe pump and pressed by the pump to control and maintain the flow rate. The printing solution was delivered through a plastic tube to the nozzle connected to the power supply, and an electric field was applied between the nozzle and target (ground) filled with and not filled with an ethanol medium. Fig. 1(a) shows the general EHDP printing process without using ethanol as a target medium, and the charged solution was printed on the target plate, as shown in the optical and SEM images (P-scaffold). As a target medium, ethanol was filled to about 3 mm in height, and a charged initial jet (the mixture of cellulose and α-TCP) was then burst into the micro/nano-fibrous bundles when immersed in the ethanol owing to the replacement of an electrospun solvent (acetone, 25.2 mN m^−1^ at 20 °C) of a α-TCP/cellulose solution, with a relatively low surface tension of the ethanol (22.10 mN m^−1^ at 20 °C).^28^ A latticed scaffold (EHDP-scaffold) consisting of this mixture was printed through the movement of the nozzle using a three-axis robot moving system.

After fabricating the scaffolds, they were immersed in PBS for 24 h for cementation of α-TCP. Through this process, the chemical composition of α-TCP changed into calcium deficient hydroxylapatite (CDHA; (3α-Ca_3_(PO_4_)_2_ + H_2_O → Ca_9_(HPO_4_)_5_(OH))).

### Comparison of composite structures fabricated using electrospinning and EHDP processes

**Fig. 2:**
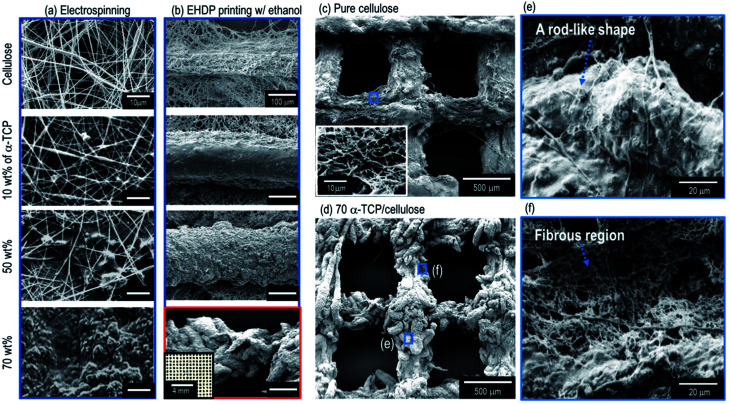
SEM images of (a) electrospun and (b) EHDP-processed structures using pure cellulose and 10 wt%, 50 wt%, and 70 wt% α-TCP. Mesh structures fabricated through the EHDP process using (c) pure cellulose and (d) 70 wt% α-TCP/cellulose. Magnified images of the strut with (e) a rod-like shape and (f) a fibrous region.

Along with the biocompatible material, the porous structure of a scaffold may be a highly important factor in fabricating ideal biomedical scaffolds.^30^ In particular, the pore structure can influence the mechanical strength and cellular activities, including the cell-seeding efficiency, cell-proliferation, and ingrowth toward the thickness direction of the scaffold. In particular, the size of the pores required in bone tissue regeneration have been suggested to be 325 ± 50 μm.^31^ However, as shown in the SEM image of the electrospun cellulose nanofibers (Fig. 2(a)), the average pore size (3.16 ± 1.02 μm) of the fibrous mat was much smaller than that of the required size.

In addition, to observe the effects of α-TCP on the pore structure of the electrospun composites, various weight fractions (10, 50, and 70 wt%) of α-TCP in the cellulose were added (Fig. 2(a)). As shown in the SEM images, as a higher weight fraction of α-TCP was added into the cellulose to improve the osteogenesis of the cellulose fibers, the pore structure of the electrospun composite (cellulose/a-TCP) gradually collapsed. In particular, for the high fraction of α-TCP (70 wt%), the pores in the electrospun mat were not observed.

**Fig. 3:**
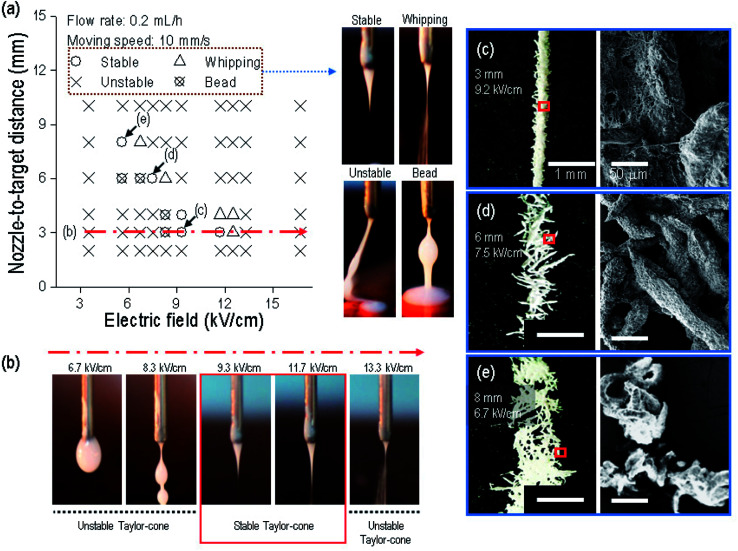
(a) Processing window of the correlation between the electric field (6.7–16.7 kV cm^−1^) and nozzle-to-target distance (3–10 mm) under a fixed flow rate of 0.2 mL h^−1^ and moving speed of 10 mm s^−1^. Optical images of fabricating conditions of an unstable jet, a beaded jet, a whipping jet, and a stable jet. (b) Optical images of an initial jet extruded from the nozzle tip for various electric fields (6.7–13.3 kV cm^−1^) under fixed parameters. Optical and SEM images of struts drawn from (c) 3, (d) 6 and (e) 8 mm nozzle-to-target distance with 9.2, 7.5, and 6.7 kV cm^−1^, respectively.

However, when using the EHDP printing process with an ethanol medium, the macropore structure of the composite, which consists of struts (the mixture of cellulose fibers and α-TCP) shown in the SEM images in Fig. 2(b), was well manipulated (see the optical image in Fig. 2(b)), which is independent of the weight fractions of α-TCP. In addition, unlike a pure cellulose mesh structure (Fig. 2(c)), a new sized rod-like structure (a mixture of cellulose fibers and α-TCP) with a diameter of about 50 μm was observed in the high weight fraction (70 wt%) of the α-TCP composite (Fig. 3(d and e)). The composite strut consisted of a rod-like structure (Fig. 2(e)) and cellulose fibers (Fig. 2(f)), respectively. We believe a structure assembled using rod-like shapes of the EHDP-processed composite may be obtained based on the fact that the repulsion from the electrostatic forces may be insufficient owing to the high viscosity of the mixture (70 wt% of α-TCP/cellulose). Fig. 2(c and d) shows SEM images of the mesh structure of the pure cellulose scaffold and EHDP-processed composite structure with 70 wt% of α-TCP, indicating a completely different morphological structure.

### Processing conditions for fabricating α-TCP/cellulose composite

It is difficult to obtain composite scaffolds that have a high weight percent (over 70 wt%) of bioceramics owing to the low processability of bioceramics and the severe post-treatment conditions required to sustain a complex pore structure. Therefore, biocomposites having a high volume fraction have become a manufacturing issue that needs to be overcome.^32^ In addition, because the vol% of inorganic elements in most human bones might be above 55 vol%, we fixed the fraction of α-TCP in the cellulose composite as 70 wt% in this study.

In general, the EHDP process using an ethanol medium can be influenced by various processing parameters such as the electric field, nozzle-to-target distance, flow rate, and nozzle moving speed.^33^ These parameters should be adequately adjusted to print different composite materials into a 3D fibrous structure, and thus in the present study, we tried to validate the adequate printing conditions using a α-TCP/cellulose composite.

To proceed with the parameter study, we fixed the size of the nozzle at 21G, and the weight fraction of α-TCP at 70 wt%. We then found the possible printing range of the electric field and the nozzle-to-target distance because these two parameters have a greater effect on the fabrication of a fibrous structure.^28^Fig. 3(a) shows that a fibrous strut consisting of α-TCP/cellulose was formed within a very narrow range of the electric field for each nozzle-to-target distance under fixed conditions (flow rate of the mixed solution of 0.2 mL h^−1^, and nozzle moving speed of 10 mm s^−1^). Specifically, within a 3 mm distance, the composite solution did not reach the target below 8.3 kV cm^−1^ owing to the insufficient strength of the electric field (Fig. 3(b)). At 8.3 kV cm^−1^, the solution was ejected toward the target, but an initial straight non-homogeneous jet with beads was observed, indicating that the electric field was still sufficiently low to generate a stable Taylor-cone and the initial jet. However, from 9.3 to 11.7 kV cm^−1^, a stable initial jet was observed, which created a straight strut. At 13.3 kV cm^−1^, the printing solution was scattered owing to the overly high electric field strength, and thus the solution was whipped, similar to an electrospinning process. Through the processing diagram, we can confirm that the electric field range used to fabricate a stable initial jet can be merely 9.3 to 11.7 kV cm^−1^ at a 3 mm nozzle-to-target distance.

From the process diagram shown in Fig. 3(a), we can select the proper electric field in each nozzle-to-target distance to obtain a stable Taylor-cone at the nozzle tip. To observe the strut morphology for each set (selected electric field strength at the nozzle-to-distance, namely, 9.3 kV cm^−1^ at 3 mm, 7.5 kV cm^−1^ at 6 mm, and 6.7 kV cm^−1^ at 8 mm), we tested the formation of a single-line strut, shown in Fig. 3(c–e). As shown in the optical and SEM images, although the struts were printed on the straight-line under the conditions of 7.5 kV cm^−1^ at 6 mm and 6.7 kV cm^−1^ at 8 mm owing to the stable Taylor-cone, the rod-like structure in the printed strut was scattered, whereas for the condition of 9.2 kV cm^−1^ at 3 mm, the stable formation of an aggregated strut was achieved, and thus we fixed the nozzle-to-target distance and electric field as 3 mm and 9.2 kV cm^−1^ to fabricate the composite mesh structure.

**Fig. 4:**
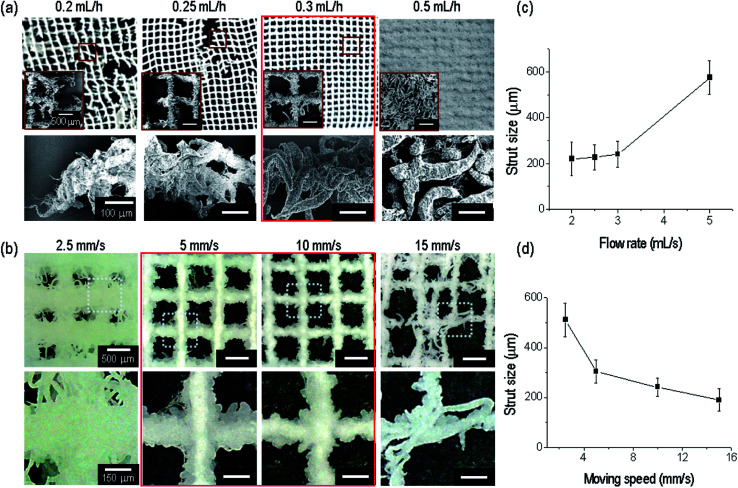
(a) Optical and SEM images of a lattice fibrous structure for various flow rates (0.2–0.5 mL h^−1^) under fixed parameters (nozzle-to-target distance of 3 mm, electric field of 9.7 kV cm^−1^, and nozzle moving speed of 10 mm s^−1^). (b) Optical images of a lattice structure for various nozzle moving speeds (2.5–20 mm s^−1^) under fixed parameters (nozzle-to-target distance of 3 mm, electric field of 9.7 kV cm^−1^, and flow rate of 0.3 mL h^−1^). Strut size based on each (c) flow rate and (d) moving speed.

The relation between the nozzle-to-target distance and electric field was evaluated based on the fabrication of a stable strut formation. Under a fixed distance (3 mm) and electric field condition (9.3 kV cm^−1^), appropriate printing conditions (the nozzle moving speed and flow rate) should be selected to build a continuous fibrous mesh structure with macropores. With a fixed nozzle-to-target distance and under an electric field, we varied the flow rate from 0.2 to 0.5 mL h^−1^. The flow rates (0.2 and 0.25 mL h^−1^) were insufficient to combine the strut-to-strut well into the entire mesh structure (Fig. 4(a)). However, for a flow rate of 0.3 mL h^−1^, the formation of the mesh structure was even owing to the stable bond between struts. At over 0.5 mL h^−1^, the pores in the mesh structure disappeared owing to an overly high flow rate of the solution, and thus we selected a flow rate of 0.3 mL h^−1^.

In addition, the nozzle moving speed was varied to evaluate the formation of a mesh structure having a controllable pore size with previously fixed processing conditions (electric field of 9.3 kV cm^−1^, nozzle-to-target distance of 3 mm, and a flow rate of 0.3 mL h^−1^). As shown in Fig. 4(b), a proper range of the nozzle moving speed to form a porous mesh structure was about 5 to 10 mm s^−1^.

In addition, the size of the printed strut size at the flow rate and moving speed was analyzed. The strut size was gradually increased with an increase in the flow rate (Fig. 4(c)), and decreased with an increase in the moving speed (Fig. 4(d)), as expected.

Through an evaluation of the previous processing conditions for the successful formation of a controllable ceramic-based mesh structure, we can select the processing conditions, namely, an applied electric field of 9.3 kV cm^−1^, flow rate of 0.3 mL h^−1^, distance between the nozzle tip and the surface of target medium of 3 mm, and a nozzle moving speed of 10 mm s^−1^.

### Fabrication and characterization of composite scaffolds

**Fig. 5:**
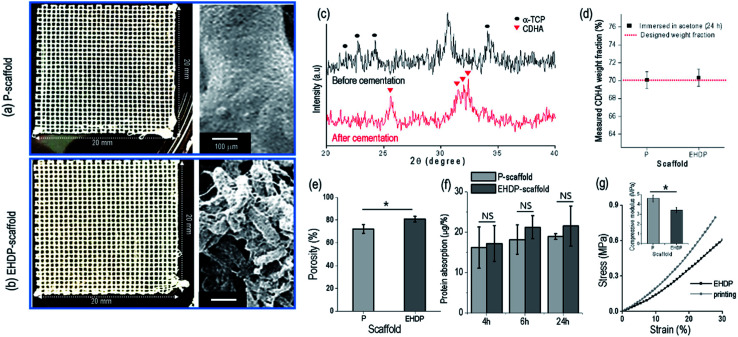
Optical and magnified SEM images of fabricated (a) P-scaffold and (b) EHDP-scaffold. (c) XRD data of the EHDP-scaffold showing different crystal peaks of α-TCP and CDHA before and after cementation of the scaffold, respectively. (d) Weight fraction of the remnant CDHA after dissolving cellulose in acetone for 24 h. (e) Porosity and (f) protein absorption divided by each porosity of the P-scaffold and the EHDP-scaffold. (g) Compressive stress–strain curves and inset figure showing the compressive moduli.

In this work, we used a control to observe the effect of the struts, which are fibrous or non-fibrous, on the cellular activities. Fig. 5(a and b) shows the P-scaffold and EHDP-scaffold using α-TCP/cellulose (7 : 3 w/w). The printed-scaffold (P-scaffold) was fabricated under the same fixed parameters as those used for fabricating the EHDP-scaffold, but the target medium (ethanol) was not used. Therefore, the charged initial jet extruded from the nozzle was directly deposited onto the ground plate, resulting in non-fibrous struts (Fig. 5(a)). The sizes of the strut and pore were measured as 259.6 ± 52.4 and 308.6 ± 41.0 μm, respectively. In addition, the EHDP-scaffold was obtained using the EHDP process under the same fixed processing conditions. An SEM image of the scaffold's surface demonstrates its fibrous structure (Fig. 5(b)). The EHDP-scaffold revealed a strut size of 243.6 ± 44.2 μm and a pore size of 298.6 ± 38.8 μm.

Fig. 5(c) shows XRD data indicating that α-TCP in the EHDP-scaffold was hydrolyzed into CDHA, which is a bioceramic similar to the inorganic components in human bones, after cementation in a PBS solution. The pattern peaks at 23–25°, 30–31°, and 34°, which correspond to the orthorhombic crystal structure of α-TCP, were rarely observed after the cementation process (24 h).^34^ Instead, new XRD peaks at 31–32° corresponding to CDHA peaks were generated. Based on this result, we can see that the cementation process may be sufficient to the hydrolysis of α-TCP.

To observe the weight fraction of CDHA in the fabricated composite scaffolds, we measured the weight fraction of the inorganic component from each scaffold after the cementation process. The cellulose was fully dissolved in acetone for 24 h, and after dissolving, the remnant inorganic component was weighted (Fig. 5(d)). There was no significant difference between the P-scaffold (70.1 ± 0.9%) and the EHDP-scaffold (70.3 ± 1.0%), which indicates that the weight fraction of the embedded bioceramic component was completely similar in each scaffold.

As shown in Fig. 5(e), the porosity of the composite scaffold was measured, and the EHDP-scaffold (80.6 ± 2.4%) revealed a higher porosity than the P-scaffold (72.1 ± 3.9%). The pore size of the lattice structure in each scaffold was comparable; however, the fibrous bundle structure of the EHDP-scaffold struts made the scaffold more porous.

The protein absorption of a scaffold ominously influences the cell attachment at the initial stage owing to the initial attachment of certain proteins (fibronectin, immunoglobulins, vitronectin, and fibrinogen).^35^ As shown in Fig. 5(f), the ability of protein absorption was normalized with each porosity, and the absorption was slightly increased along the immersed time regardless of the surface morphology of the scaffold. However, the absorption ability of the P- and EHDP-scaffolds showed no significance owing to the similar chemical compositions of the composites.

Fig. 5(g) shows the compressive stress–strain curves and moduli of the P-scaffold and EHDP-scaffold. The compressive modulus of P-scaffold (4.60 ± 0.34 MPa) shows higher than that of EHDP-scaffold (3.40 ± 0.27 MPa). Usually, it has been accepted that both the compressive strength and the Young's modulus are affected by porosity of the structure.^36–38^ Therefore, more porous struts of EHDP-scaffold resulted in lower compressive modulus. In addition, since the compressive moduli of both scaffolds were relatively low compare to those of cortical and trabecular bone tissues, the enhancement of mechanical property of the EHDP-scaffold would be our next overcoming issue.

### *In vitro* cellular activities of bioceramic scaffold

**Fig. 6:**
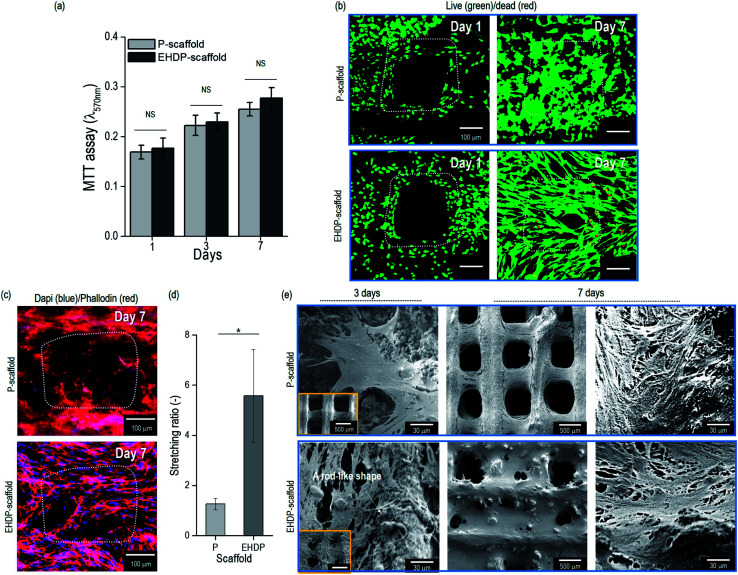
(a) MTT results after cell culture of 1, 3, and 7 days. (b) Live/dead images of P-scaffold and EHDP-scaffold after culturing for 1 and 7 days. (c) Combined DAPI/phalloidin staining images after cell culture for 7 days. (d) Measurement of stretching ratio (SR) for the P- and EHDP-scaffolds on day 7 of the cell culture. (e) SEM images of cell morphology of scaffolds for 3 and 7 days.

The preosteoblast cells (MC3T3-E1) were seeded onto the P- and EHDP-scaffolds to observe their *in vitro* cellular activities. The cell proliferation was evaluated for 1, 3, and 7 days of culturing (Fig. 6(a)). The cell proliferation was non-significant between the P- and EHDP-scaffolds for all culture days owing to the hydrophilic nature of the composites and the similar protein absorption capability. A live/dead assay was applied to indicate live cells in green, and dead cells in red, at days 1 and 7 (Fig. 6(b)). The images from day 1 show a macropore and the distributed/surrounded cells in each composite. Despite the different structures, the scaffolds demonstrated a high cell viability of over 94%, which was maintained throughout the 7 days of culturing. Therefore, this indicates that the increasing proliferation was well supported with the biocompatible cellulose and CDHA materials. In addition, the proliferated cells (at day 7) in the EHDP-scaffold, which are shown in the live/dead and nuclei (blue)/F-actin (red) images in Fig. 6(c), showed a significantly spreading/stretched surface morphology compared to those of the P-scaffold. The cell morphology was analyzed using the stretching ratio (SR), which was defined as the ratio of the longest/shortest lengths of the actin. Fig. 6(d) demonstrates the SR of the actin, and the EHDP-scaffold provides a clearly elongated morphological shape owing to the rod-like cellulose/ceramic structure shown in Fig. 2(d and e). Previous works have demonstrated that osteoblasts can be highly affected by the surface morphology of the scaffolds, and the cells, which were elongated through micropatterned surfaces, induced a highly mineralized extracellular matrix (ECM) compared to that of the cells, which were not elongated on a flat surface.^39,40^ Because of its unique morphological surface structure, we can estimate that the EHDP-scaffold can provide a platform inducing a higher mineralization of the ECM. Morphological assessment of MC3T3-E1 cells attaching to the surface of scaffolds was performed on 3 and 7 days after culturing on each scaffold (Fig. 6(e)). After 3 days of culture, the cells on P-scaffold showed a multi-polar stretching shape while the cells on EHDP-scaffold were uniaxially stretched along the rod-like shape. After 7 days of culture, cells on both P-scaffold and EHDP-scaffold proliferated well and covered the surface of scaffold, and these results correspond to the cell morphology shown in Fig. 6(b).

**Fig. 7:**
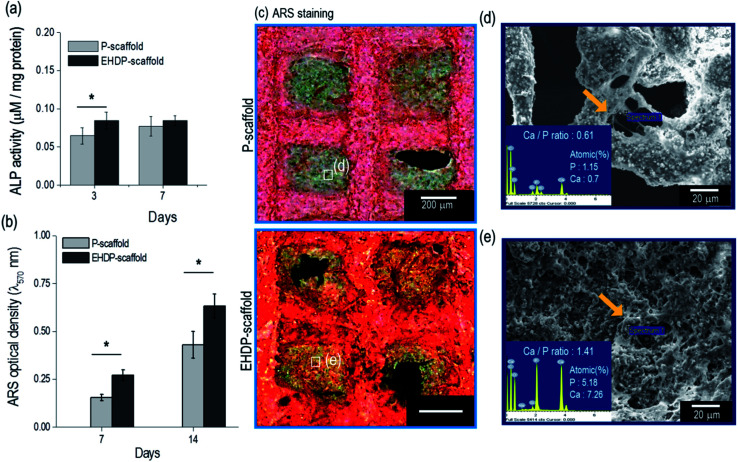
Measurement of (a) alkaline phosphate (ALP) activity and (b) relative calcium deposition. (c) Optical image of Alizarin Red S (ARS) stained scaffolds at day 7. SEM and atomic percent (Ca and P) from (d) P-scaffold and (e) EHDP-scaffold at day 7.

To observe the early stage of osteogenic differentiation, the alkaline phosphate (ALP) activity was used, and the data were normalized based on the total protein content (Table 1). The ALP activity of the EHDP-scaffold on day 3 was higher compared to that of the P-scaffold (Fig. 7(a)). However, on day 7, the activity was non-significant between the scaffolds because this is an early stage of differentiation. Moreover, the calcium deposition was quantitatively measured using Alizarin Red S (ARS) staining (Fig. 7(b)). The EHDP-scaffold revealed a much higher value of calcium deposition compared to the P-scaffold, and qualitatively, the optical images of the stained scaffolds were measured, indicating that the differentiated cells turned to red. As expected, the color was much denser in the EHDP-scaffold than in the P-scaffold (Fig. 7(c)). In addition, we measured the amounts of elemental calcium (Ca) and phosphorus (P), which were detected from the pore region of the P- and EHDP-scaffolds (Fig. 7(d and e)), respectively. As shown through the atomic percent, the calcium and phosphorus were higher in the EHDP-scaffold than in the P-scaffold. Moreover, the ratio (1.41) of Ca/P in the EHDP-scaffold was slightly less than the 1.67 of hydroxylapatite, whereas that of the P-scaffold was about 0.61. This indicates that EHDP-scaffold consisting of a unique CDHA/cellulose microfibrous surface structure provides preferable micro-environmental conditions for the cells, which induces effective cell mineralization.

**Table 1:** Total protein contents of the scaffolds on days 7 and 14

Scaffold	P-scaffold	EHDP-scaffold
Day 7	33.6 ± 5.4 mg	35.2 ± 5.8 mg
Day 14	91.0 ± 6.3 mg	93.0 ± 1.3 mg

## Conclusion

In this study, a new bioceramic scaffold consisting of α-TCP/cellulose was fabricated using an electrohydrodynamic printing process. Various processing windows were demonstrated to observe the effects of the nozzle-to-target distance, electric field, nozzle moving speed, and flow rate of the solution on the stable formation of a fibrous strut. Based on an evaluation, the most appropriate values of the parameters were selected to obtain a stable 3D fibrous structure. As a control, a non-fibrous printed 3D composite structure fabricated using the same chemical compositions, and with a similar pore and strut size, was used. Although the protein absorption and cell proliferation were completely similar between the control and experimental group, the osteogenic activities of ALP and calcium mineralization were significantly higher in the experimental group, indicating that the suggested fibrous cellulose/ceramic composite has high potential as a bioceramic scaffold for a bone tissue regeneration platform.

## Conflicts of interest

There are no conflicts to declare.

## Supplementary Material

RA-008-C8RA03657H-s001
