# Supplementary material for: Biomimetic cellulose/calcium-deficient-hydroxyapatite composite scaffolds fabricated using an electric field for bone tissue engineering
Source: RSC Adv. 2018 Jun 6;8(37):20637–47. doi: 10.1039/c8ra03657h (PMC9080802; doi:10.1039/c8ra03657h)
Supplement: RA-008-C8RA03657H-s001 [file RA-008-C8RA03657H-s001.pdf]

Electronic Supplementary Material (ESI) for RSC Advances.
This journal is © The Royal Society of Chemistry 2018

Supporting Information

Biomimetic cellulose/calcium-deficient-hydroxyapatite composite scaffolds fabricated using an electric field for bone tissue engineering

MyoJin Kim[1], MiJi Yeo[1], Minseong Kim, and GeunHyung Kim[*]

*Department of Biomechatronic Eng., College of Biotechnology and Bioengineering, Sungkyunkwan University (SKKU), Suwon, Korea

*Corresponding author: e-mail: gkimbme@skku.edu, Tel.: +82-31-290-7828

Maximum size of the fabricated scaffold

The maximum size of the fabricated scaffold was 25 × 25 × 2 mm^3 under the stable printing condition of 9.7 kV/cm electric field, 3 mm nozzle-to-collector distance, and 0.3 mL/h flow rate (Fig. S1). Here, the maximum height of the scaffolds was about 2 mm because of the accumulated residual charge on the surface of scaffold and the repulsion force. The combination of residual charge and repulsion force may cause the instability of electrical charge when the struts were deposited layer-by-layer, and consequently decreased the stacking accuracy over 2 mm height [1]. On the other hand, the fabricated width and length of the scaffold, 25 × 25 mm^2, was the maximum dimension for the collector used as a target. If the larger size of the collector was provided as a printing equipment, it would be extended with larger scale work.

1. B. Zhang, J. He, X. Li, F. Xu and D. Li, *Nanoscale*, 2016, **8**, 15376-15388.

Figure

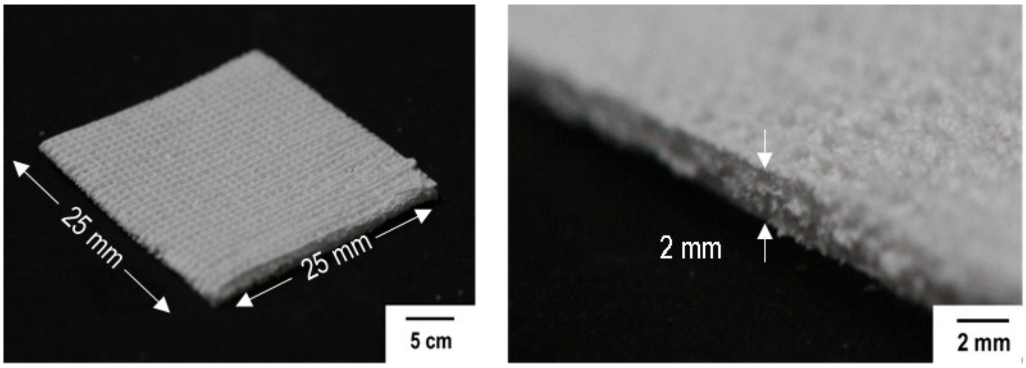

Fig. S1. 5 layered scaffold fabricated in maximum size ($25 \times 25 \times 2$ mm^3).
